# Adaptive Kalman Filtering Localization Calibration Method Based on Dynamic Mutation Perception and Collaborative Correction

**DOI:** 10.3390/e27040380

**Published:** 2025-04-03

**Authors:** Zijia Huang, Qiushi Xu, Menghao Sun, Xuzhen Zhu, Shaoshuai Fan

**Affiliations:** 1 National Key Laboratory of Multi-Domain Data Collaborative Processing and Control, Xi‘an 710068, China; beiyouhzj@163.com; 2 State Key Laboratory of Networking and Switching Technology, Beijing University of Posts and Telecommunications, Beijing 100876, China; 1518668454@bupt.edu.cn (Q.X.); 2024110214@bupt.cn (M.S.); fanss@bupt.edu.cn (S.F.)

**Keywords:** adaptive Kalman filtering, dynamic mutation perception, collaborative correction

## Abstract

Aiming at the problem of reduced positioning accuracy of unmanned swarm navigation systems due to dynamic abrupt noise in a complex electromagnetic environment, this paper proposes an adaptive Kalman filtering positioning and calibration method based on dynamic mutation perception and collaborative correction. This method optimizes the performance of Kalman filtering by monitoring the mutation of acceleration and velocity in real time, designing a dynamic threshold detection mechanism, adaptively adjusting the covariance matrix, and using multidimensional scaling analysis to calculate the similarity of trajectories and collaboratively correct the current state. The experiment uses simulation and real scene data and compares algorithms such as the traditional extended Kalman filter to verify the effectiveness of the proposed method, providing an effective solution for the collaborative positioning of an unmanned swarm under complex electromagnetic interference.

## 1. Introduction

In the modern electromagnetic countermeasure environment, the reliability and accuracy of the navigation and positioning systems of unmanned swarms are challenged by complex electromagnetic interference [1,2,3]. Electromagnetic interference not only affects the normal operation of the systems but also more easily induces dynamic errors. Such errors usually manifest as mutations and are difficult to predict, making it hard for traditional positioning systems to maintain accurate and stable state estimation in complex environments. Especially in autonomous driving [4] and military applications [5], precise navigation capabilities are of vital importance. The abrupt errors caused by electromagnetic interference require the navigation systems to quickly identify and adjust in order to ensure the stability and accuracy of the systems. Particularly during the execution of tasks, when unmanned units in a group are faced with such interference, their motion states change suddenly and unpredictably. These abrupt motion states translate directly into significant increases in velocity and acceleration errors. Meanwhile, unmanned swarms, with their great potential for coordinated and distributed operations, are composed of multiple interconnected unmanned units that are envisioned to perform complex tasks in a variety of scenarios. However, the current navigation and positioning algorithms for unmanned bee colonies still have significant shortcomings. The existence of complex interference, as well as the need for seamless cooperation between group members, makes the existing algorithms insufficient, resulting in poor performance and limited scalability in practical applications.

When dealing with these challenges, the combination of a global navigation satellite system (GNSS) and the inertial measurement unit (IMU) has become the mainstream solution [6,7,8]. The GNSS provides precise positioning information on a global scale, while the IMU achieves independent navigation by measuring acceleration and angular velocity. However, GNSS signals are vulnerable to electromagnetic interference and obstruction, resulting in signal interruptions or increased errors. On the other hand, when used for an extended period, IMU will also cause position drift due to cumulative errors. To address these issues, the Kalman filter (KF) [9], as an efficient data fusion algorithm, has been widely applied in integrated GNSS and IMU navigation. By fusing the observation data from the GNSS and IMU, the KF dynamically updates the state estimation and error covariance, thereby improving the positioning accuracy and the system’s robustness in the presence of noise and uncertainties. Nevertheless, in the face of abrupt noise and dynamic errors, the performance of the traditional KF still has limitations, which has prompted researchers to explore more adaptive filtering methods [10].

Current unmanned swarms navigation and positioning algorithms still face numerous limitations. Traditional approaches, including those based on KF, generally presuppose a relatively ideal environment. They inadequately account for the intricate and diverse interference characteristics prevalent in real-world scenarios. The mutation noise disrupts the precision of both the observation and state models in these filtering algorithms. For instance, in the case of the extended Kalman filter (EKF), linearization errors occur when dealing with nonlinear systems, and this can be exacerbated by the presence of complex interference. In the unscented Kalman filter (UKF), although it attempts to better approximate nonlinear distributions compared to EKF, sudden and unpredictable interference can still lead to inaccurate sigma point selection and subsequent incorrect state estimations. In addition, collaborative correction mechanisms in existing studies are often oversimplified and lack adaptability. Many methods rely on fixed-parameter models for cooperative correction, which cannot adapt to dynamic changes of environment and the behavior of unattended clusters.

Dynamic mutation perception is of vital importance in this context. It can help the system identify sudden state changes and make timely adjustments to reduce error accumulation. However, relying solely on dynamic mutation perception may not completely eliminate errors. Therefore, it is necessary to introduce a collaborative correction mechanism. This paper proposes an adaptive Kalman filtering method that combines dynamic mutation perception and collaborative correction. By monitoring the dynamic changes in acceleration and velocity data in real time, the covariance matrix is adaptively adjusted when there are state mutations, thus optimizing the performance of the Kalman filter. At the same time, multidimensional scaling (MDS) [11] is used to calculate the similarity between trajectories, and the velocity state of the current trajectory is corrected by using the velocity information of adjacent trajectories. This method can not only effectively deal with sudden state changes but also significantly improve the positioning accuracy and stability of the system through collaborative correction.

The structural arrangement of this paper is as follows: In the second part, an overview of the existing navigation and positioning calibration methods and their limitations is provided. The third part elaborates in detail on the adaptive Kalman filtering method proposed in this paper, including the dynamic mutation perception and the collaborative correction mechanism. The fourth part presents the experimental setup and result analysis, verifying the effectiveness of the proposed method. Finally, the fifth part summarizes the research achievements and proposes the future research directions.

## 2. Related Work

For the collaborative positioning and calibration of unmanned swarms, currently, common positioning and calibration methods include the integration of GNSS and IMU [12], the integration of visual and inertial sensors [13], UWB-based positioning [14], as well as SLAM technology [15], etc. [16]. The integration of GNSS and IMU employs Kalman filtering technology to offer high-precision positioning. Nevertheless, in complex environments, GNSS signals are prone to interference. The integration of visual and inertial sensors is applicable in environments without GNSS coverage, but it is sensitive to lighting variations and has high computational complexity. UWB technology enables high-precision positioning, but it necessitates the additional installation of base stations. SLAM achieves self-positioning by constructing an environmental map and is suitable for dynamic environments, but it demands a relatively high amount of computational resources. These methods perform satisfactorily in specific scenarios. However, when confronted with complex and changeable environments, they still have limitations. This paper precisely focuses on the approach of data fusion and improves the Kalman filtering method to achieve more accurate positioning and calibration.

The Kalman filter collaborative positioning algorithm is the most widely applied information fusion technology at present and is mainly used in linear systems. Since the observation model of the unmanned platform is a nonlinear model, the performance of the Kalman filter will significantly decline, leading to an increase in positioning errors. As a result, researchers have developed nonlinear filtering algorithms such as EKF [17], UKF [18], and the cubature Kalman filter (CKF) [19]. Additionally, the interacting multiple-model Kalman filter (IMM-KF) [20] has emerged as a powerful alternative in handling systems with multiple possible models. IMM-KF switches between different Kalman filter models based on a Markov chain, enabling it to adapt to various system dynamics more effectively. In the context of unmanned swarms, where the operating environment can be highly unpredictable, IMM-KF has the potential to better account for different motion patterns and environmental conditions. To address these issues, researchers have proposed the adaptive extended Kalman filter (AEKF) [21,22]. By adjusting the filtering parameters in real time to adapt to the changes in the system model and noise characteristics, the filtering performance in the case of model mismatch is improved. Some studies achieve the optimization of dynamic environments by adaptively adjusting the noise covariance matrix. These methods can improve the filtering accuracy and the system’s robustness to a certain extent and are especially suitable for the dynamic collaborative positioning of multi-unmanned swarm systems.

In addition, positioning correction methods based on the data collaboration of other unmanned platforms have also been deeply studied. Through sharing location information and sensor data [23], multi-unmanned platform systems can achieve collaborative calibration and correction of positioning errors [24]. For example, in the method based on relative measurement, communication and ranging technologies among unmanned platforms are used to measure the relative distances between them [25]. By using multiple sets of relative distance measurement values, combined with known initial positions or reference points, geometric constraint relationships are constructed, and optimization algorithms such as the least squares method [26] are used to correct the GNSS positions of each unmanned platform. There are also some methods that utilize visual feature matching and shared map data [27]. Through the collaborative perception among unmanned platforms, a more accurate environmental modeling can be achieved, thereby further calibrating the positioning information. Moreover, collaborative calibration methods based on deep learning are gradually emerging [28]. By learning the error patterns and sensor characteristics from historical data, these methods can effectively reduce positioning errors in dynamic and complex environments [29,30]. On this basis, the key research of this paper lies in calculating the similarity between trajectories and using the velocity information of adjacent trajectories to correct the velocity state of the current trajectory. By identifying the similar trajectories of multiple unmanned platforms and optimizing the velocity of the current unmanned platform using their velocity patterns, the overall positioning accuracy and the robustness of the system can be improved.

## 3. Materials and Methods

**Figure 1:**
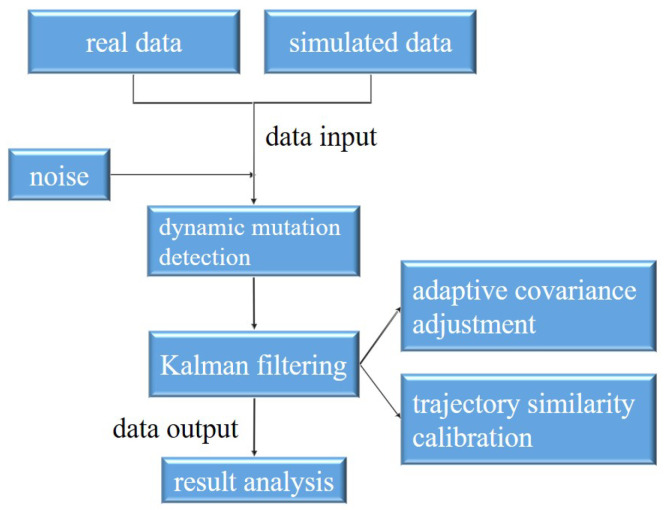
A data processing system in which real data and simulated data, together with noise as input, are first detected by dynamic mutation and then adjusted by adaptive covariance and calibrated by trajectory similarity; finally, the output data are analyzed.

In this section, we detail the materials and methods employed in our research. Our goal is to develop a comprehensive approach for enhancing the navigation and state estimation of unmanned swarms in complex environments. The following subsections describe the key techniques we use, including dynamic mutation detection, noise modeling, adaptive covariance adjustment, and trajectory similarity calibration. As shown in Figure 1, the system flow chart includes dynamic mutation detection, noise input, a filtering process, and so on.

### 3.1. Dynamic Mutation Detection

In our research, we aim to accurately model and account for the behavior of unmanned systems in complex environments. As for the causes of such sudden changes in speed and acceleration, in our study, these changes are mainly due to, for example, changes in the motion of an unmanned system while avoiding sources of interference. The same is true for adjustments to speed and acceleration noise, which we perform to better simulate the uncertainties that these evasive operations bring. In the estimation of the motion state, in order to accurately identify the mutation in acceleration and velocity, this study proposes a mutation detection method based on a dynamic threshold. Considering the high correlation between velocity and acceleration, in the dynamic mutation detection method, we analyze the rate of change of acceleration and velocity simultaneously. This method detects possible mutation points by analyzing the rates of change of acceleration and velocity in real time and combining the state differences between the previous and current moments. The specific steps are as follows:

First, calculate the rates of change of acceleration and velocity. Given each time step *k*, calculate the rates of change of the current acceleration and velocity. The formulas are as follows:

(1) $$\Delta a=\left|\right|a_{k}-a_{k-1}\left|\right|,$$

(2) $$\Delta v=\left|\right|v_{k}-v_{k-1}\left|\right|,$$

Then, adaptive adjustment is performed. When a mutation point is detected, the current motion state is re-evaluated, and the dynamic thresholds $tau_{a}$ (for acceleration) and $tau_{v}$ (for velocity) are updated through the smoothing factor β to adapt to the new motion pattern and ensure a rapid response to subsequent state changes. The smoothing factor β (where 0 < β < 1) is a parameter used for adjusting the dynamic threshold. A smaller β value gives more weight to historical data, while a larger β value emphasizes the current data.

(3) $$\tau_{a}=\beta \tau_{a}+\left(1-\beta \right)\Delta a,$$

(4) $$\tau_{v}=\beta \tau_{v}+\left(1-\beta \right)\Delta v,$$

Finally, perform the judgment of mutation. At each time step, calculate the difference between the current state and the state at the previous moment. If the difference exceeds the set threshold, it is determined as a mutation point. The parameters $\lambda_{a}$ and $\lambda_{v}$ are coefficient factors used for mutation judgment. They are usually determined based on prior knowledge of the motion characteristics of the system or through experimental calibration.

(5) $$is\_abrupt\_change=\left(\Delta a>\lambda_{a}\tau_{a}\right)\vee \left(\Delta v>\lambda_{v}\tau_{v}\right).$$

### 3.2. Noise Model

In order to enhance the robustness of the state estimation, this study introduces a state-related Gaussian noise model into the acceleration, velocity, and GNSS data. Especially when there are mutations in the motion state, the noise level will be dynamically adjusted to reflect the uncertainties of the environment.

Firstly, the basic noise model is described as follows: The initial noise model is based on the characteristics of each sensor, assuming that the noise follows a zero-mean Gaussian distribution. Considering that the positioning error caused by GNSS interference is usually not Gaussian (for example, GNSS interference may cause signal fading, so that the positioning error presents the characteristics of Rayleigh distribution), we adopt a mixed distribution model for positioning error.

(6) $$n_{a}∼N\left(0,\sigma_{a}^{2}\right);n_{v}∼N\left(0,\sigma_{v}^{2}\right);n_{gnss}∼0.5N\left(0,\sigma_{gnss}^{2}\right)+0.5Rayleigh\left(\sigma_{r}\right),$$

Next, the noise is adjusted according to the relevant state. When a mutation is detected, the noise level increases dynamically based on the amplitude of the state change. This measure aims to reduce the short-term errors caused by the mutation and improve the accuracy of the state estimation.

(7) $$\sigma_{a}^{\prime }=\beta_{a}\sigma_{a};\sigma_{v}^{\prime }=\beta_{v}\sigma_{v};\sigma_{gnss}^{\prime }=\beta_{gnss}\sigma_{gnss};\sigma_{r}^{\prime }=\beta_{r}\sigma_{r};.$$

Here, $\beta_{a}$, $\beta_{v}$, and $\beta_{gnss}$ are state-related amplification factors that are dynamically adjusted according to the degree of mutation. The determination of the β value takes into account the characteristics of the system motion and the expected amplitude of the state change. For example, in a system with relatively stable motion, where we expect only small, gradual changes in the noise properties, we would choose a small beta value. In contrast, if the system operates in a highly dynamic environment with frequent and significant state changes, we will choose a larger beta value that highlights the current state change and can adapt more quickly to new noise conditions. Rapid changes in speed and acceleration are often due to external interference and other factors, which will increase the uncertainty of sensor measurement in reality. For example, the evasive motion makes the motion state of the unmanned units suddenly change and increases the noise of each sensor. Then, the adjusted noise is injected into the acceleration, velocity, and GNSS data, enabling the Kalman filter to better handle state mutations.

### 3.3. Adaptive Covariance Adjustment

In the state update process of the Kalman filter, this study adopts an adaptive adjustment strategy based on dynamic mutation perception to dynamically adjust the covariance matrix.

First, set the system state vector $x_{k}$. It includes the platform’s position $p_{k}$, velocity $v_{k}$, and acceleration $a_{k}$, namely $x_{k}={\left[\begin{array}{ccc} p_{k} & v_{k} & a_{k} \end{array}\right]}^{T}$.

In the state prediction process, the Kalman filter conducts the state prediction and covariance prediction at the current moment based on the state estimation $x_{k}$ at the previous moment and the state transition matrix **F**, and $F=\left[\begin{array}{ccc} I_{3} & \Delta tI_{3} & 0.5\Delta t^{2}I_{3}\\ 0_{3} & I_{3} & \Delta tI_{3}\\ 0_{3} & 0_{3} & I_{3} \end{array}\right]$. Here, **F** is the Jacobian matrix of the state transition function f(·):

(8) $$x_{k+1}=f\left(x_{k},u_{k}\right)+w_{k},$$

(9) $$P_{k+1}=F_{k}P_{k}{F_{k}}^{T}+Q_{k},$$

In the update process, the innovation is first calculated using the actual observation value $z_{k}$ and the predicted state $x_{k}$. Here, **H** is the observation matrix, and $H=\left[\begin{array}{ccc} I_{3} & 0_{3} & 0_{3} \end{array}\right]$, which is used to map the state to the observation space:

(10) $$y_{k}=z_{k}-Hx_{k},$$

The innovation covariance matrix $S_{k}$ is used to measure the uncertainty of the prediction: $S_{k}=HP_{k}H^{T}+R$, where R is the measurement noise covariance matrix.

In order to improve the adaptability of the filter to mutation and noise, an adaptive covariance adjustment technique is adopted. The innovation $y_{k}$ and the innovation covariance $S_{k}$ are used to calculate the adaptive factor $\alpha_{k}$:

(11) $$\alpha_{k}=\frac{y_{k}^{T}y_{k}}{tr(S_{k})},$$

Formula (11) aims to measure the relative size of the innovation value relative to innovation covariance, $y_{k}^{T}y_{k}$ measures the degree of innovation deviation, and $tr(S_{k})$ reflects the overall uncertainty of innovation. The predicted covariance matrix is adjusted according to the adaptive factor to enhance the system’s robustness against mutation and noise. The min function here is used to prevent $\alpha_{k}$ from being too large or too small:

(12) $$P_{k}=min\left(1/\alpha_{k},1\right)P_{k},$$

Formula (12) reduces $P_{k}$ when $\alpha_{k}$ is large, making the filter more sensitive to new measurements, and avoids overreaction when $\alpha_{k}$ is small and $P_{k}$ is constant. The Kalman gain $K_{k}$ determines the extent to which the observation value affects the state update:

(13) $$K_{k}=P_{k}H_{k}^{T}{S_{k}}^{-1},$$

Correct the predicted state according to the innovation and the Kalman gain to obtain the updated state estimation:

(14) $${\hat{x}}_{k}=x_{k}+\lambda K_{k}y_{k},$$

The factor λ in Equation (14) is a weighting factor, and setting λ to <1 makes the filter more conservative when incorporating innovation into state estimates. Finally, update the state covariance matrix $P_{k}$ to reflect the uncertainty of the new state:

(15) $$P_{k+1}=\left(I-K_{k}H_{k}\right)P_{k}.$$

### 3.4. Trajectory Similarity Calibration

In unmanned swarms, units’ coordinated motion generates valuable data, like relative velocity and acceleration correlations. These data offer crucial information for trajectory correction within the swarm. The idea of trajectory similarity calibration is to utilize the correlated data generated by the coordinated motion of each unit in an unmanned swarm. Due to the coordinated motion among the units, these trajectories exhibit a certain degree of similarity. To improve the overall accuracy of trajectory estimation, this study employs the MDS method to calculate the similarity between trajectories and corrects the current trajectory using the velocity information of adjacent trajectories.

The MDS method is used to analyze the velocity states of multiple trajectories and calculate the similarity distance matrix between them. First, the distance matrix is calculated as follows:

(16) $$D_{i,j}=\mu_{v}\left|\right|{\hat{v}}_{i}-{\hat{v}}_{j}\left|\right|+\mu_{a}\left|\right|a_{i}-a_{j}\left|\right|,$$

The $\mu_{v}$ and $\mu_{a}$ in Formula (16) are weight factors, which are used to balance the importance of velocity difference and acceleration difference in calculating similar distances $D_{i,j}$. Here, both are simply set as 0.5. In the similarity calibration process, we utilize the $\hat{v}$, which is obtained from state estimation. The distance matrix **D** is centered to obtain the centered matrix **B**:

(17) $$B=-\frac{1}{2}JD^{2}J,$$

where $J=I-\frac{1}{n}11^{T}$, I is the identity matrix, and **1** is the all-ones matrix. Regarding $11^{T}$, its main function is to centralize the distance matrix **D**.

(18) $$B=V\Lambda V^{T},$$

where **V** is the matrix of eigenvectors, and Λ is the matrix of eigenvalues. Select the largest eigenvalue and its corresponding eigenvector to calculate the final one-dimensional coordinate X:

(19) $$X=\Lambda \sqrt{V[1]},$$

For each trajectory *j*, calculate the distance weight with other trajectories, and normalize these weights:

(20) $$\omega_{i}=\left\{\begin{array}{c} \frac{1}{|X_{i}-X_{j}|},\begin{array}{cc} if & i\neq j \end{array}\\ \sum_{k\neq j}\omega_{k},\begin{array}{cc} if & i=j \end{array} \end{array}\right.,$$

(21) $$\omega_{i}=\omega_{i}/sum\left(\omega \right),$$

Finally, conduct the velocity correction. Based on the weights of each trajectory, select the velocity information of adjacent trajectories, calculate the weighted average velocity, and correct the velocity state of the current trajectory:

(22) $${\hat{v}}_{j}=\frac{{\hat{v}}_{j}+\sum_{k!=j}\omega_{k}\cdot {\hat{v}}_{k}}{2}.$$

### 3.5. The Implementation Details

When implementing the adaptive Kalman filtering localization calibration method based on dynamic mutation perception and collaborative correction, the virtual data and real data are collected first. Professional simulation software is used to generate virtual inertial measurement data, including acceleration, speed, and other information, and corresponding virtual positioning data. At the same time, a specific inertial navigation and positioning integrated module is used to collect real acceleration, speed, and positioning data in the actual scene and ensure the synchronization of the two types of data. Then, according to the pre-set noise model, Gaussian noise is added to the acceleration, speed, and positioning data in accordance with the actual situation to simulate the interference factors in the real environment. Then, we enter the core process of adaptive Kalman filtering, that is, adjusting the covariance matrix with dynamic mutation sensing and using the trajectory similarity to correct the velocity state. Finally, the experiment will compare the performance of this method with other filtering algorithms, and comprehensively evaluate the advantages of this method in positioning accuracy and anti-interference ability by analyzing the positioning errors and the performance of the data processed by each algorithm.

## 4. Results

### 4.1. Source of Experimental Data

Virtual Simulation Data: The IMU data are generated by the simulation software GNSS-INS-SIM (Version 2.1) for virtual simulation data. It also simulates the GNSS signals to construct a GNSS/IMU integrated navigation environment. The simulation data can provide high-precision motion state information and allow testing of the performance of the filtering algorithm under different noise conditions.

**Figure 2:**
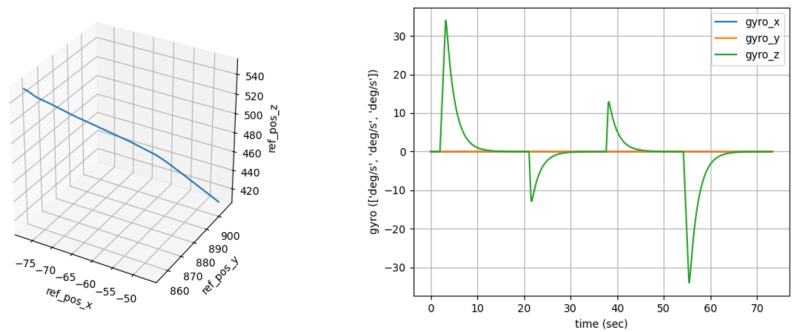
The generation of virtual simulation data. On the left is the reference position curve in 3D space. The trend of the curve presents specific spatial trajectory changes, reflecting the position movement in 3D space. The figure on the right shows the gyroscope data over time, which helps to analyze the motion state of the object during motion.

**Figure 3:**
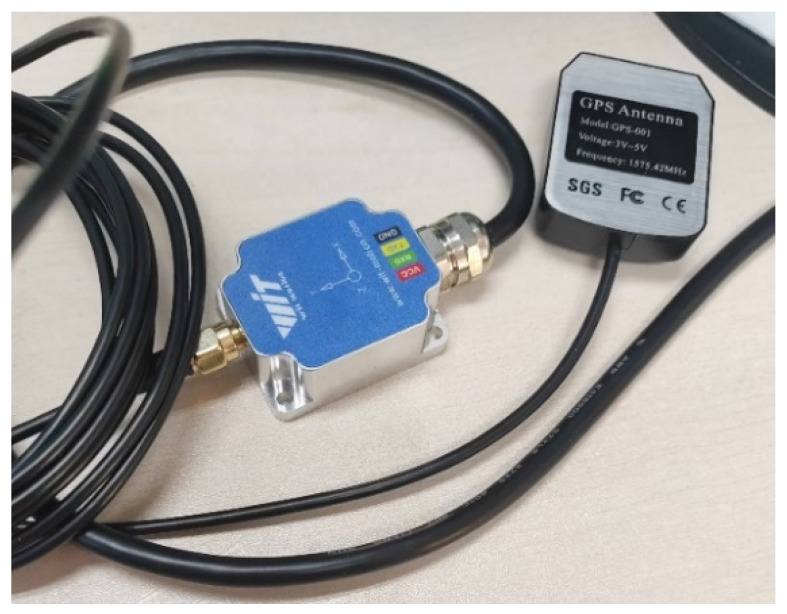
The real data measurement module (sourced from WitMotion Shenzhen Co., Ltd., Shenzhen, China), which is composed of Beidou and inertial navigation and simultaneously collects latitude and longitude information and motion information during movement.

Real Measurement Data: The WTGAHRS3-232 Beidou + inertial navigation integrated navigation module is used to collect actual motion data, including acceleration, angular velocity, GNSS position data, etc., and record the motion trajectory of the unmanned or mobile platform in the real environment. In the experiment, the speed is directly output by the inertial navigation module, and the experiment simulates the speed of a low-speed 1–5 m/s unmanned platform performing low-altitude monitoring tasks. The following Figure 2 and Figure 3 show the measurement module used.

**Figure 4:**
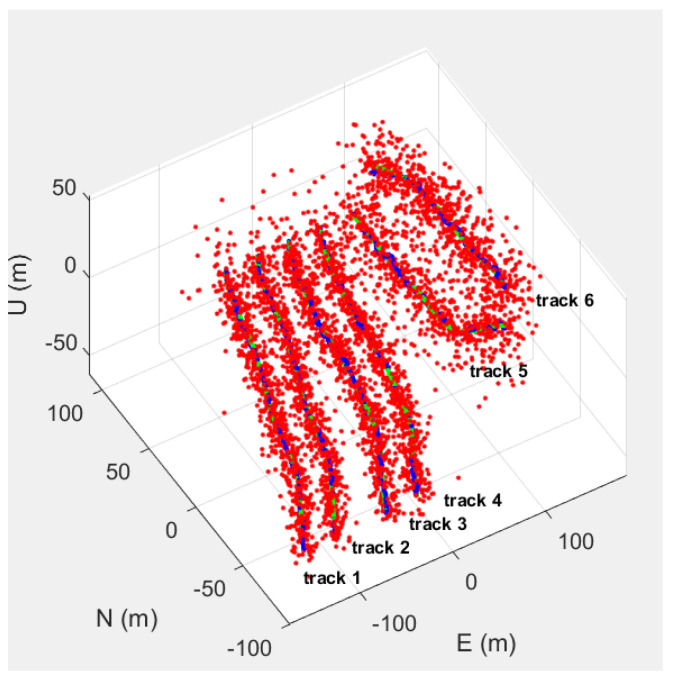
The comparison of experimental trajectories. This coordinate system is an east-north-up (ENU) coordinate system. The observation data prior to filtering are denoted by red scatter points. The error-free data are illustrated by the green trajectory. Meanwhile, the blue trajectory represents the final estimated trajectory refined by the method proposed in this paper.

Experimental Scene Setup: The virtual experimental scene is based on the GNSS-INS-SIM simulation environment. Different motion trajectories are set up, and random noise is introduced to test the adaptability of the algorithm. In the real experimental scene, in an open environment, the inertial navigation and GNSS data of the WTGAHRS3-232 integrated navigation module are collected to evaluate the effect of the method in practical applications. Figure 4 shows the trajectory data of the experiment. Among these, trajectories 1, 2, 3, and 4 are simulated trajectories generated by GNSS-INS-SIM, while trajectories 5 and 6 are the real trajectories collected by the WTGAHRS3-232. In our simulation data, the mutations in the trajectories are mainly due to different motion stages. When moving from one stage to another, the motion parameters of the two stages are very different, which leads to the abrupt change of the trajectory. We generate the ground truth with a separate, high-precision differential positioning module. The system has a positioning accuracy of 0.4 m level and 0.8 m elevation, which is enough for our truth reference. The red scatter points represent the GNSS observation data that have not been filtered, the green trajectory represents the data without errors, and the blue trajectory is the final estimated trajectory optimized by the method in this paper. The similarity calibration in our study is indeed applied continuously. Irrespective of whether the trajectories are highly similar (like tracks 1–4) or more different (such as tracks 5 and 6), we maintain the calibration process. When the motion states between trajectories differ significantly, the weight assigned to similarity-based adjustments is automatically reduced. This design ensures that our system can leverage the similarity information when it is relevant while minimizing the interference of dissimilar data. The key objective of this experiment is to verify the effectiveness of the dynamic mutation perception and collaborative correction mechanism proposed in this paper by comparing the trajectory results of different filtering methods.

### 4.2. Experimental Methods

This experiment conducts data collection, filtering processing, and result analysis through the following steps. Firstly, in the data collection stage, GNSS-INS-SIM is used to generate simulated IMU and GNSS data. At the same time, real navigation data, including IMU and GNSS positioning information, are collected through the WTGAHRS3-232. All data are time-synchronized for subsequent comparative analysis. In the data preprocessing stage, outliers in the GNSS data are removed using a statistical method. We calculate the mean and standard deviation of the GNSS position data over a short time window, and the latitude and longitude coordinates are transformed to ensure the uniformity of the data format. In terms of recording conditions, real-world data were collected on clear days with no significant weather disturbances and deployment in open areas. Meanwhile, according to the noise model, a state-related Gaussian noise model is introduced into the acceleration, velocity, and GNSS data. In the adaptive Kalman filtering processing stage, a dynamic mutation perception mechanism is combined with a collaborative correction method. The similarity of trajectories is utilized to optimize the velocity state, and a comparative experiment is carried out with traditional algorithms such as the EKF. Finally, in the experimental result analysis stage, the error between the filtered trajectory and the real trajectory is calculated. The adaptability and accuracy of different methods in the case of mutation are evaluated, and the performance of different filtering methods is compared.

In experiments, parameter setting is crucial to accurately evaluate algorithm performance. We set the noise parameters as $\sigma_{a}^{2}=0.1\left|a\right|$, $\sigma_{v}^{2}=0.2\left|v\right|$, $\sigma_{gnss}^{2}=5$, $\sigma_{r}^{2}=1$, and we use β values of 1.1–1.3 in the stable scene and 1.4–1.6 in the dynamic scene. In dynamic mutation detection, scale factors $\lambda_{a}=2$ and $\lambda_{v}=3$ are used to judge mutation. In trajectory similarity calibration, the weight factors $\lambda_{a}$ and $\lambda_{v}$ for distance calculation are set to 0.5.

### 4.3. Experimental Result Evaluation

#### 4.3.1. Noise and Mutations

**Figure 5:**
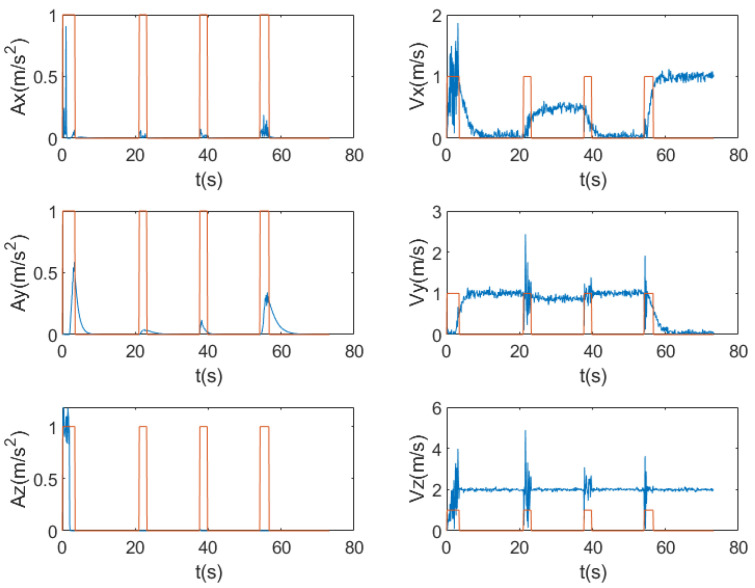
The changing states of acceleration and velocity under the influence of noise, the blue lines respectively showing that the accelerations (Ax, Ay, Az) in the x-, y-, and z-axis directions have obvious fluctuations at specific time points, and the velocity curves (Vx, Vy, Vz) are complex in fluctuation and also change significantly at corresponding moments. Meanwhile, the orange rectangular-framed parts in the figure represent the detected time instants when the states undergo mutation.

**Figure 6:**
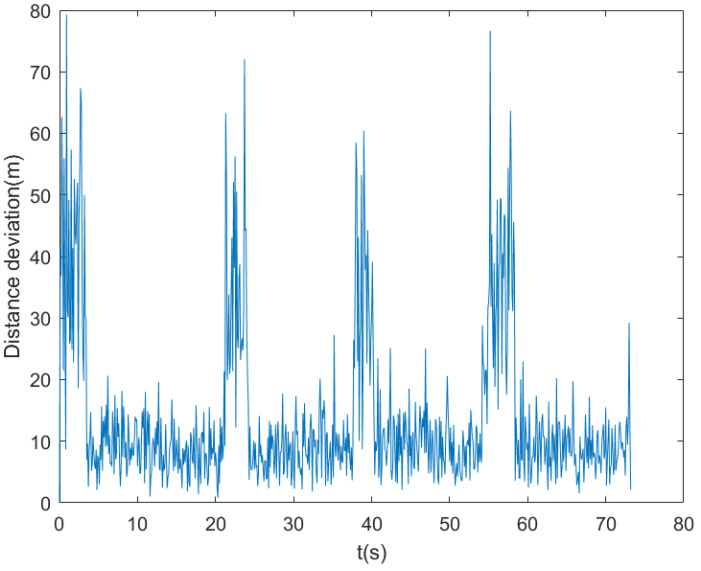
The positioning error curve under the influence of noise. The horizontal axis represents time, and the vertical axis represents the positioning error. It can be clearly seen from the figure that the positioning error curve exhibits significant fluctuations. At certain specific moments (corresponding to the mutation points of the system state), the positioning error increases sharply.

This experiment analyzes the variations of velocity, acceleration, and position errors; detects the mutation points in the system; and analyzes the trend of noise changes. Figure 5 shows the variation curves of the velocity and acceleration of trajectory 1. As can be seen from the figure, at certain moments, there are obvious mutations in the velocity and acceleration, indicating that the system state has undergone drastic changes at these time points. These mutations may be caused by environmental interference, sensor errors, or external excitations. At the mutation points, the noise level usually increases significantly. Therefore, it is necessary to specially process the data at these moments to reduce their impact on the filtering effect. Figure 6 shows the position error curve. It can be observed from the figure that at the mutation points, the position error fluctuates greatly, and the error value increases significantly. The occurrence of these mutation points has a great impact on the positioning accuracy of the navigation system. Meanwhile, the large variation range of the noise near the mutation points further indicates the instability of the system during mutation.

#### 4.3.2. Comparison of Algorithm Results

**Figure 7:**
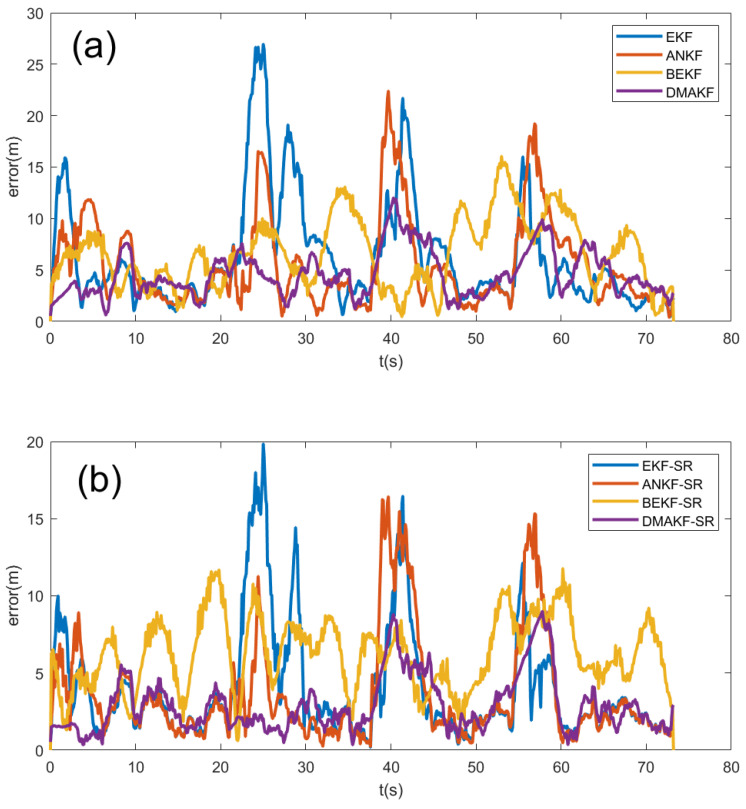
The positioning errors under different algorithms: (**a**) shows the results without similarity correction, from which it can be seen that the DMAKF has the best effect; (**b**) shows the results of combining each algorithm with similarity correction, and it can be seen that the overall effect after adding similarity fusion is better than that in (**a**).

In order to evaluate the performance of different filtering methods, this experiment compares the EKF, the adaptive noise adjustment Kalman filter (ANKF) [31], the bidirectional extended Kalman filter (BEKF) [32], the dynamic mutation adaptive Kalman filter (DMAKF), and the effect of combining the above methods with similarity correction (SR). The comparison indicators mainly include the position error of each trajectory and the average position error. Table 1 below lists the average position errors of different filtering methods, and “raw GNSS” represents the deviation of the unprocessed GNSS data from the true position, which can be considered as the reference standard for comparison with the estimated positions from different filtering algorithms. Figure 7 shows the influence of different algorithms on the position error of the same trajectory.

The experimental results show that the standard EKF method can maintain a certain estimation accuracy when the noise is small, but it has a large error at the mutation points, and it is difficult to quickly respond to environmental changes. The ANKF improves the adaptability of the filtering to mutations by adaptively adjusting the noise covariance, reducing the error to a certain extent. The BEKF performs better by optimizing the filter structure and reducing the error. In contrast, after detecting the mutation points, the DMAKF can dynamically adjust the covariance matrix, significantly improving the stability of the filtering when facing mutations. After further combining with similarity correction, the filtering effects of all methods are improved. It can not only suppress the noise more effectively but also further improve the filtering results. The experimental data show that the DMAKF-SR method is superior to other methods in terms of the accuracy of position estimation. For the influence of mutation points, it has the smallest error, and the trajectory is closest to the real value. Overall, the DMAKF-SR scheme combines the advantages of adaptive mutation adjustment and similarity correction. Specifically, we can more intuitively see the performance difference between these algorithms when we observe the curve in the provided graph at the point of abrupt change. For EKF, the curve shows a sharp jump at the abrupt point, indicating a wide range of fluctuations in error. Although the ANKF curve does not change as dramatically as the EKF curve, there is still a significant deviation from the ideal value at these critical points. BEKF smooths the curve to a certain extent, but there are still significant deviations compared to DMAKF. The DMAKF curve has the smallest deviation at the abrupt point, which is due to its dynamic covariance regulation mechanism. This is consistent with the analysis that DMAKF has the smallest error and the highest accuracy in this challenging situation. Compared with the standard EKF method, its average positioning error is reduced by 56.13%, and its robustness is significantly enhanced, demonstrating the best filtering effect. However, it is important to note that the DMAKF-SR algorithm has some limitations. Its performance relies on accurate mutation point detection and effective similarity calculation. When the data contain significant errors or anomalies, such as sensor malfunctions causing incorrect acceleration or velocity values, the mutation detection mechanism may misjudge, leading to improper covariance matrix adjustments. Meanwhile, uncorrelated data can distort the trajectory similarity calculations, making the velocity correction based on these similarities less reliable. This can ultimately affect the algorithm’s ability to handle mutations effectively and degrade the final filtering results.

## 5. Conclusions

In this paper, aiming at the problems of positioning accuracy and stability of unmanned swarm navigation systems in complex electromagnetic environments, an adaptive Kalman filtering method combining dynamic abrupt change perception and collaborative correction is proposed. This method dynamically adjusts the filtering by detecting the abrupt changes in acceleration and velocity in real time and combines with the trajectory similarity correction mechanism, which effectively enhances the system’s adaptability to abrupt states and improves the navigation accuracy and robustness.

This method holds great significance in practical applications. It can be applied to various tasks, like tracking and positioning drone swarms. It ensures precise navigation in complex electromagnetic confrontation scenarios, which boosts combat effectiveness and mission success rates. For transportation, it suits autonomous vehicles in intelligent traffic systems. In urban areas where tall buildings cause obstructions and complex electromagnetic signals generate strong noise interference, this method enables vehicles to accurately determine their positions, thus enhancing driving safety and reliability. Regarding logistics, it is applicable to drone delivery services. It allows drones to achieve accurate positioning in complex environments, ensuring efficient cargo delivery.

The experimental results show that the EKF has a large error when facing abrupt states, and it is difficult to quickly adapt to environmental changes. The ANKF and the BEKF improve the estimation accuracy to a certain extent, but it is still difficult to fully cope with drastic state changes. In contrast, the DMAKF method proposed in this paper can effectively reduce the position error at the abrupt change points, and it can further optimize the state estimation by combining with the trajectory similarity correction, achieving more stable and accurate navigation and positioning. In both simulation data and real data tests, this method shows superior anti-interference ability and adaptability.

The main contribution of this study is to propose a dynamic abrupt change perception mechanism. By analyzing the abrupt changes in the motion state in real time, the adaptability of the filter to sudden errors is improved. At the same time, a trajectory similarity correction mechanism is introduced, which optimizes the velocity state by using the similarity relationship between the trajectories of multiple unmanned platforms and improves the overall positioning accuracy of the system. Through the verification of simulation and measured data, the effectiveness of the method proposed in this paper in complex dynamic environments is proved, providing a highly robust and accurate positioning calibration scheme for unmanned swarm navigation. In future research, it is possible to further explore the combination of deep learning and data-driven methods to optimize the abrupt change detection and noise modeling and to improve the intelligence level of adaptive filtering. The multi-sensor fusion strategy can be studied, and auxiliary information such as vision and lidar can be introduced to enhance the navigation ability in different environments. For large-scale unmanned swarm systems, the collaborative correction method can be optimized to improve the real-time performance and computational efficiency of multi-vehicle collaborative positioning, and so on.

## Figures and Tables

**Table 1:** This table presents the trajectory positioning errors of different algorithms. The average errors of traditional EKF, ANKF, and BEKF are 6.4385 m, 5.9638 m, and 4.7868 m, respectively. The proposed DMAKF algorithm shows excellent error control in tracks 1–6, with an average error of only 3.729 m, significantly lower than other algorithms. With similarity correction, DMAKF-SR further reduces the average error to 2.8246 m, the lowest among all. This shows that both DMAKF and DMAKF-SR outperform the others in accuracy, enhancing positioning reliability.

Filtering Method	Track 1 (m)	Track 2 (m)	Track 3 (m)	Track 4 (m)	Track 5 (m)	Track 6 (m)	Average Error (m)
Raw GNSS	13.2924	13.6172	13.2395	13.6411	28.7547	28.8997	18.5741
EKF	4.7594	5.3409	5.0611	4.779	9.3826	9.3078	6.4385
ANKF	4.5449	4.5755	4.3646	4.6321	8.9453	8.7206	5.9638
BEKF	4.0199	3.7964	3.464	3.734	7.0759	6.6305	4.7868
DMAKF	3.0298	2.3564	2.3853	1.9402	5.3241	7.3379	**3.729**
EKF-SR	3.8294	3.9638	4.0171	3.8161	5.3223	5.3824	4.3885
ANKF-SR	3.8437	3.8839	3.5971	3.75	4.808	5.2778	4.1934
BEKF-SR	3.3222	2.5491	3.6061	2.9658	4.8313	5.328	3.7671
DMAKF-SR	2.4504	1.8537	2.1792	1.8073	4.2274	4.4297	**2.8246**

## Data Availability

The data used in this study are available from the corresponding author upon reasonable request.
